# Dramatic Response to Pramipexole in Delayed-Onset Parkinsonism from Osmotic Demyelinating Syndrome

**DOI:** 10.5334/tohm.66

**Published:** 2020-06-16

**Authors:** Steve C. Han, Linn Katus, Steven Frucht

**Affiliations:** 1 Department of Neurology, NYU Grossman School of Medicine, US; 2 Department of Neurology, New York-Presbyterian Hospital/Columbia University, US

**Keywords:** Parkinsonism, extrapyramidal symptoms, osmotic demyelinating syndrome

## Abstract

**Background:**

Delayed parkinsonism and dystonia are recognized phenomena in osmotic demyelinating syndrome (ODS). Dopamine receptor agonists and levodopa have been reported to benefit select patients.

**Case report:**

We report a patient with ODS with severe pseudobulbar deficits, parkinsonism and dystonia, poorly responsive to levodopa, who experienced a remarkable improvement with pramipexole.

**Discussion:**

A marked response to pramipexole with lack of response to levodopa suggests a pre-synaptic source for his deficits coupled with injuries to non-nigral compensatory structures.

**Highlights:**

This case highlights a dramatic response of osmotic demyelination-induced parkinsonism/dystonia to pramipexole. A lack of response to levodopa suggests deficits in the pre-synaptic nigral as well as non-nigral compensatory structures.

## Introduction

Osmotic demyelinating syndrome (ODS) occurs as a result of overly rapid correction of hyponatremia [1]. Depending on the locations of the lesion and clinical manifestations, ODS may be separated into two categories: central pontine myelinolysis (CPM) and extrapontine myelinolysis (EPM). CPM is typically associated with severe quadriparesis, bulbar palsy, coma or locked-in state. It may also be associated with dysarthria, dysphagia, ophthalmoplegia or facial paresis [1]. EPM is characterized by altered consciousness, confusion, emotional lability, ataxia, tremor, myoclonus, akinetic mutism, catatonia, dysautonomia, quadriparesis, dystonia, choreoathetosis or parkinsonism [1].

While magnetic resonance imaging (MRI) is helpful in evaluating suspected ODS, the findings may take several weeks to become apparent [1]. Typical brain MRI findings include T2 and FLAIR hyperintensities as well as T1 hypointensities without contrast enhancement [1]. It is important to note that the extent of MRI signal intensity does not necessarily correlate with clinical outcome [2,3]. In addition, these signal changes may eventually resolve, which make MRI less reliable overtime [2].

This case report presents a patient presenting with pseudobulbar deficits, parkinsonism and dystonia from ODS, whose symptoms were resistant to levodopa but markedly responsive to pramipexole.

## Case Description

**Figure 1:**
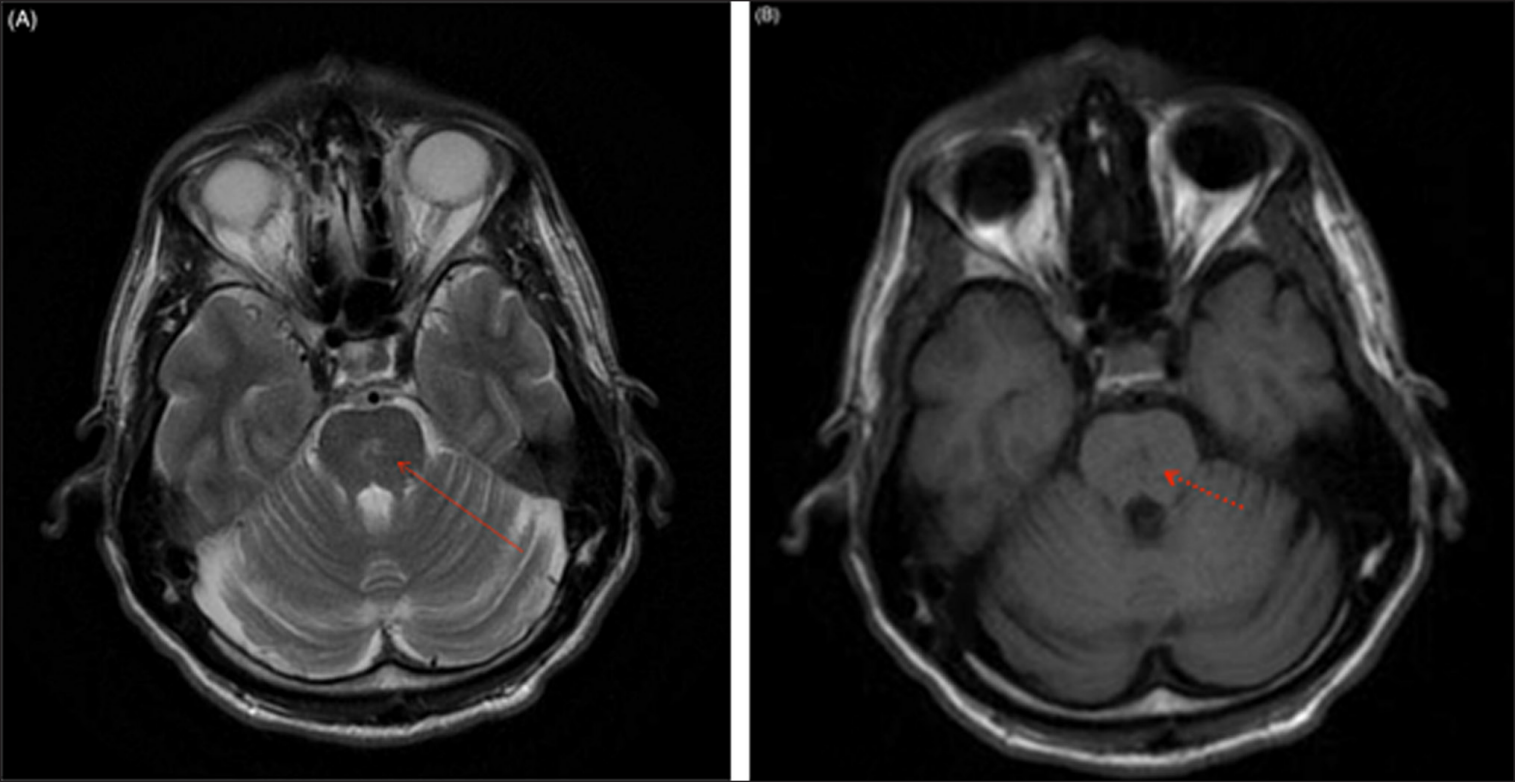
**Neuroimaging in ODS. (A)** Axial T2-weighted brain MRI of the patient showing hyperintensity of the central pons (solid arrow) and **(B)** Axial T1-weighted brain MRI of the patient showing hypointensity of the central pons (dotted arrow).

**Figure 2:**
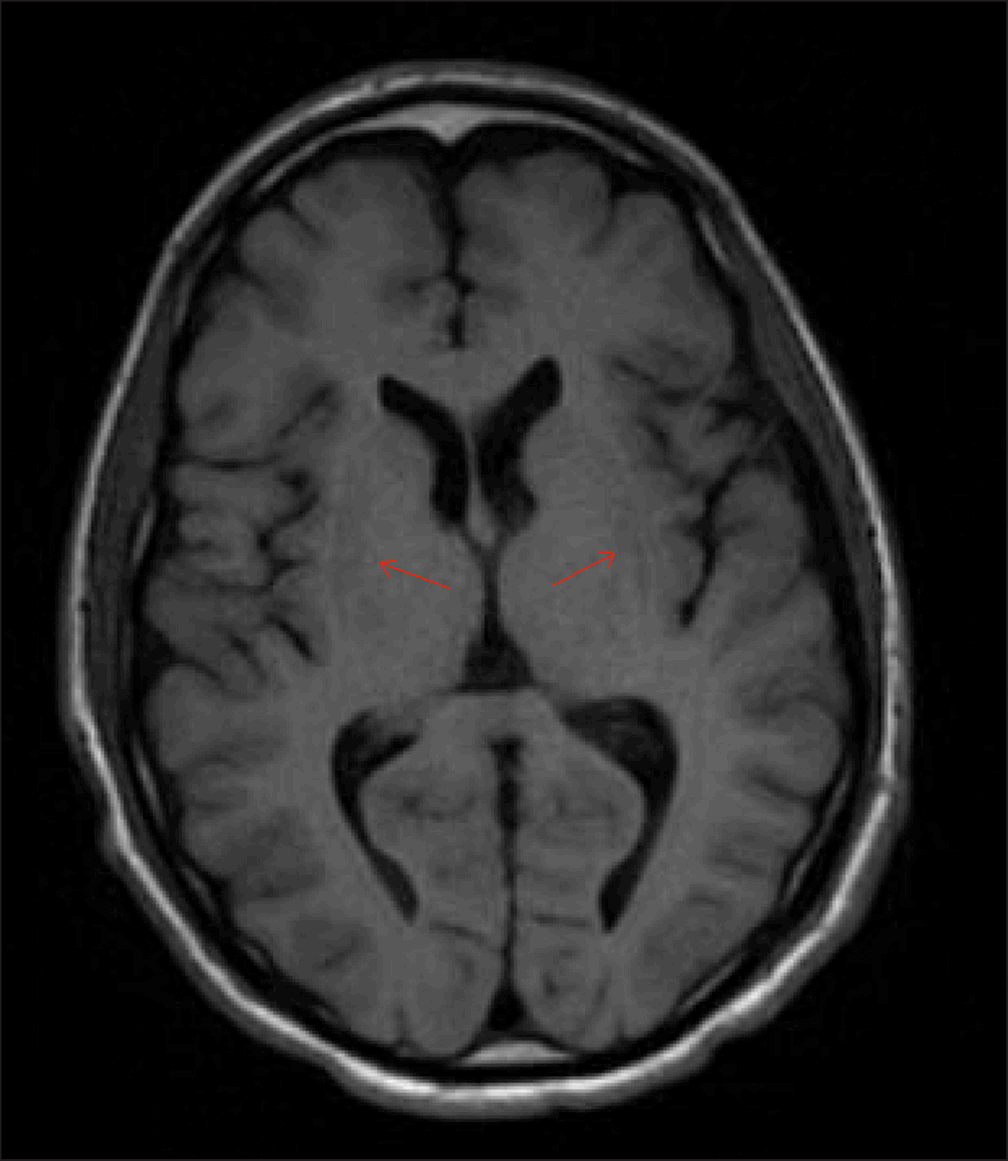
**Neuroimaging in ODS.** Axial T1-weighted brain MRI of the patient showing hypointensities of the bilateral lentiform nuclei (red arrows).

A 42-year-old man with history of von Willebrand disease type 1 was prescribed desmopressin after a recent nasal septoplasty in the setting of epistaxis post-surgery. Within days of discharge, patient presented to the hospital with a seizure. His sodium at the time of presentation was 122 mEq/L which was corrected to 132 mEq/L within a period of 24 hours. After transfer to our institution, ODS was confirmed on MRI which showed T2 hyperintense lesion at the central pons as well as T1 hypointensities at the bilateral lentiform nuclei (Figures 1, 2). Hospital course was complicated by a seizure, locked-in syndrome for approximately 3 weeks, dysphagia, dysarthria and difficulty with bilateral hand coordination. He was eventually discharged to rehabilitation with some improvement of these symptoms but did not return to his baseline.

Approximately 11 months after his first admission, he was re-admitted for worsening dysphagia, dysarthria and bilateral hand discoordination. The severity of the dysphagia progressed to the point where he required a PEG placement for sustenance. The patient could not speak and required a cellular device in order to communicate. In addition to inability to swallow, he could not use his hands to write, hold a cup, type on a keyboard or write. Patient also showed bradykinesia, dystonia of bilateral hands and rigidity.

Due to these aforementioned neurological symptoms, the patient was initiated on carbidopa-levodopa 25/100 mg one tablet three times daily, subsequently increased to 1.5 tablets three times daily, without improvement. Due to worsening depression in the setting of his deteriorating health, patient attempted suicide by ingesting 20–30 tablets of carbidopa-levodopa. Despite the overdose, the patient did not subjectively notice any improvement in his neurological symptoms.

A neurological evaluation performed 1.5 years after the initial diagnosis of ODS showed mild cranial masking with facial dystonia and a risus sardonicus (Video 1). He was severely dysarthric and almost completely unintelligible. Evaluation also revealed mild square wave jerks and mild dystonic flexion posturing of the left metacarpophalangeal joints. No resting, postural or kinetic tremor was noted. Finger tapping was moderately impaired bilaterally with breakdown in the amplitude and cadence. There was mild bradykinesia of overall movements, moderately bradykinetic finger movements, and moderate bilateral cogwheeling. He had no difficulty sitting up from the chair with arms crossed and demonstrated ability to walk with good stride length but with reduced left arm swing. Pull test was negative.

**Video 1:** **Before initiation of pramipexole (only on carbidopa-levodopa).** Risus sardonicus with significant dysarthria, bradykinetic movement (low amplitude on finger tapping) and dystonia involving the hands are observed.

In the following months, amantadine 100 mg twice daily was added. The patient showed minimal benefit and was started on trihexyphenidyl 2 mg thrice daily as well as pramipexole 0.5 mg thrice daily (later titrated to 1 mg thrice daily). He showed gradual response on this regimen with improvement in his pseudobulbar palsy and facial masking. When the dose of pramipexole was increased to 1 mg three times daily there was a dramatic improvement in speech and movements. He was able to speak clearly in long sentences though the volume of speech was observed to be reduced. Facial masking and voluntary movements were markedly improved (Video 2).

**Video 2:** **On pramipexole 3 mg/day.** Significant improvement of dysarthria and risus sardonicus with greater facial expressivity are observed.

Despite the aforementioned improvement, he developed compulsive shopping behavior and sexual disinhibition three months after starting on pramipexole, leading to the discontinuation of pramipexole. Neurological examination after the discontinuation of pramipexole revealed worsening of dysarthria and parkinsonian symptoms. For treatment of these compulsive behavior, he was initially started on valproic acid and eventually transitioned to lamotrigine for mood stabilization with good effect. Transdermal rotigotine (2 mg daily) was added without significant benefit. Due to the lack of efficacy with prior regimen, pramipexole was re-introduced and he responded again to modest dose (1.5 mg daily) (Video 3).

**Video 3:** **Upon discontinuation of pramipexole.** Rotigotine was added as limited benefit with increase in other medications including levodopa, trihexyphenidyl and amantadine. Patient without noticeable improvement of dysarthria with rotigotine. Rotigotine was subsequently stopped and pramipexole was restarted and titrated up slowly on 12/2015 to 1.5 mg per day. Patient experienced improvement of dysarthria subsequently.

## Discussion

The pathophysiology of ODS is complex. Briefly, the cerebral adaptation to serum hyponatremia occurs via multiple mechanisms. The serum hypotonicity from the initial hyponatremia causes water to flow across the blood-brain barrier, increasing the brain water content as well as the intracellular water content [4]. Astrocytes lose intracellular osmolytes in response to serum hypotonicity. This allows these cells to maintain isotonicity with the serum while limiting a significant increase in intracellular water [4]. Following this cerebral adaption, the rate of correction of hyponatremia is the critical inciting factor in injury. The intracellular osmolytes in the astrocytes cannot be quickly replaced when the brain volume begins to decrease in response to a rapid correction of hyponatremia [4]. Consequently, it is surmised that the loss of intracellular water as well as the movement of potassium and sodium back into these cells cause injury and demyelination [4].

Treatment of ODS remains largely symptomatic and supportive. Treatment with thyrotropin releasing hormone, plasma exchange, methylprednisolone and intravenous immunoglobulin has not shown clear evidence of efficacy [1]. Symptomatic treatments include therapies involving the dopaminergic system with the two main classes of medications being levodopa and dopamine receptor agonists.

Levodopa supplementation allows the presynaptic neurons to release more dopamine into the synapse, resulting in greater stimulation of the dopamine receptors. In contrast, dopamine receptor agonists directly stimulate the dopamine receptors, bypassing the process of presynaptic storage and release of the neurotransmitter [5,6,7].

Review of multiple cases of movement disorder associated with ODS reveals a variable response to levodopa (Table 1). It should be noted, however, that delayed-onset parkinsonian symptoms appear to be more persistent or less responsive to therapy. Cases of parkinsonism with onset within days after diagnosis of ODS have been shown to generally respond to supportive care or levodopa (Table 1). When a few of these cases were followed up at a later time point, there were either residual parkinsonian symptoms or symptoms resistant to treatment (Table 1: Toft et al., Seah et al., Schwarz et al.). This variability in and resistance to clinical response in the delayed-onset variant may be explained by the poor reorganization and repair of neural structures over time after the initial injury [1].

**Table 1:** Review of cases of ODS presenting with extrapyramidal symptoms.

Study	Year	Case	Approximate time of symptom onset	Prominent clinical signs	Treatment	Outcome

Halimet al. [12]	2018	Case 1	4 days after presentation	Rigidity, bradykinesia, tremor, impairment of horizontal and vertical gaze	Levodopa/benserazide, trihexyphenidyl, IVIG*, dexamethasone	Improvement of symptoms
Rizviet al. [13]	2012	Case 1	4 days after presentation	Tremor, gait difficulty, bradykinesia, dysarthria, cogwheel rigidity	Levodopa-carbidopa	Improvement of symptoms
Imamet al. [14]	2012	Case 1	9 days after presentation	Bradykinesia, dysphagia, hypomimia, rigidity, spasticity	Levodopa-carbidopa	Moderate improvement of symptoms
Kwonet al. [15]	2011	Case 1	5 days after presentation	Rigidity, tremor, bradykinesia, postural instability, hypophonia, pseudobulbar palsy, autonomic dysfunction	Levodopa, anticholinergic medications, benzodiazepine	No improvement in symptoms (death from cardiac arrest 10 days after presentation)
Toftet al. [16]	2011	Case 1	28 days after presentation	Dysarthria, bradykinesia, ataxia, gait instability, dystonia	None	Persistence of symptoms
		Case 1	6 years after presentation	Tremor, cogwheel rigidity after 6 years	Levodopa, apomorphine, ropinirole	Improvement of symptoms except for mild bradykinesia of left side
Postet al. [17]	2009	Case 1	15 days after presentation	Dysarthria, bradykinesia, masked face, hypophonia, cogwheel ridigity	None	Spontaneous recovery but with mild masked face
Sajithet al. [18]	2006	Case 1	1 week after presentation	Dysarthria, ataxia, cogwheel rigidity, tremor, decreased oro-bucco-lingual movements	Levodopa-carbidopa, baclofen	Improvement of symptoms
Seahet al. [19]	2002	Case 1	9 days after presentation	Tremor, rigidity, myoclonic jerks	Supportive care	Slowly resolved except for spasticity and rigidity
		Case 1	1 month after presentation	Tremor, bradykinesia, rigidity, gait instability, dystonia, dyskinesia, choreoathetosis	Levodopa/bensarazide, trihexyphenidyl, baclofen	No improvement of movement disorder symptoms even after >1 year of follow up, with severe rigidity, bradykinesia and generalized dystonia
Kim et al. [20]	2003	Case 1	2 weeks after correction of hyponatremia	Masked face, dysarthria, bradykinesia, tremor, cogwheel rigidity, gait ataxia	Levodopa-carbidopa	Improvement of symptoms
Nagamitsuet al. [21]	1999	Case 1	2 weeks after correction of hyponatremia	Masked face, bradykinesia, dysarthria, gait disturbance, dysphagia, sialism, glossal palsy, tremor, cogwheel rigidity	Levodopa-carbidopa, amantadine	No initial improvement with amantadine but with marked improvement with levodopa
Schwarzet al. [22]	1998	Case 1	6 days after presentation	Bradykinesia, cogwheel rigidity, facial hypomimia, monotonous speech, parkinsonian gait with retropulsion	Levodopa/benserazide	Initial resolution of parkinsonian signs with levodopa/benserazide but with return of mentioned symptoms
		Case 1	4 months after presentation	Dystonia, dysphagia, spasmodic dysphonia	Tiapride, perphenazine	Improvement of dysphagia, retrocollis, oromandibular dystonia but persistent focal dystonia of the right arm and spasmodic dysphonia even after 20 months
Maraganore et al. [23]	1992	Case 1	5 months after presentation	Dystonia, dysarthria, adductor spastic dysphonia	Trihexyphenidyl, baclofen	No improvement in symptoms, exhibited movement disorder symptoms for at least 1 year
		Case 2	6 months after presentation	Tremor, forward stooped gait, dysarthria, decreased arm swing, supranuclear left horizontal conjugate gaze palsy, pseudobulbar affect	None	No improvement in symptoms, exhibited movement disorder symptoms for at least 1 year
		Case 3	5 weeks after presentation	Dystonia, masked face	None	Dystonia worsened over the next month, lost to follow up
Tisonet al. [24]	1991	Case 1	41 days after admission	Tremor, cogwheel rigidity, gait difficulty, dystonia with choreoathetosis	Trihexyphenidyl, tiapride	Improvement of symptoms

* IVIG: Intravenous immunoglobulin.

Our patient responded minimally to levodopa but showed a marked dose-dependent improvement with pramipexole. The worsening of his symptoms upon discontinuation of pramipexole and subsequent modest improvement upon re-initiation support its therapeutic effect. Another case report has also demonstrated the efficacy of pramipexole in extrapontine myelinolysis that presented in the setting of adrenal failure [8]. In this report, the patient had developed parkinsonian symptoms (diffuse rigidity with bradykinesia, hypomimia, resting tremor, festinant gait) that significantly improved with pramipexole; however, no other therapy including levodopa or other dopamine agonists were tried in this case.

The etiology of the dichotomous response in our case warrants further discussion. One explanation may be that the patient had such significant destruction of the presynaptic neurons from ODS that the amount of converted dopamine in the remaining few neurons could not result in clinically significant activation of postsynaptic dopamine receptors. Alternatively, because the postsynaptic dopamine receptors are intact, direct postsynaptic dopamine receptor agonists, such as pramipexole, could still achieve good clinical response as it bypasses the aforementioned presynaptic problem. In addition, direct dopamine agonists, with their longer half-lives than levodopa, may maintain a sustained activation of the postsynaptic dopamine receptors and result in a clinically significant response [6]. Levodopa, however, continues to show efficacy in advanced idiopathic Parkinson’s disease (PD), in which there are substantial destruction of the pre-synaptic nigral neuronal [9]. In our case, levodopa may have been less effective in treating EPS in ODS compared to idiopathic PD because ODS may have injured the basal ganglia or non-basal ganglia neurons and their pathways involved in compensatory mechanisms in idiopathic PD [10].

The lack of response to rotigotine compared to pramipexole should be discussed as both are nonergot dopamine agonists. While the mechanism is not completely understood, these dopamine agonists have different selectivity for different dopamine receptors [11]. Rotigotine shows high affinity and potency for D1, D2 and D3 receptors. Pramipexole shows high affinity and potency at D2 and D3 but preferentially stimulates D3 receptors compared to D2 receptors. While the clear role of specific receptor agonism is unclear, the differences in the receptor binding of the two agents may explain the dichotomous clinical response in this particular patient.
